# Effects of therapeutic horseback riding on post-traumatic stress disorder in military veterans

**DOI:** 10.1186/s40779-018-0149-6

**Published:** 2018-01-19

**Authors:** Rebecca A. Johnson, David L. Albright, James R. Marzolf, Jessica L. Bibbo, Hayley D. Yaglom, Sandra M. Crowder, Gretchen K. Carlisle, Amy Willard, Cynthia L. Russell, Karen Grindler, Steven Osterlind, Marita Wassman, Nathan Harms

**Affiliations:** 1 Research Center for Human Animal Interaction, College of Veterinary Medicine, University of Missouri, Columbia, MO 65211 USA; 2 Research Service, Harry S. Truman Memorial Veterans’ Hospital, Columbia, MO 65211 USA; 3 School of Social Work, University of Alabama, Tuscaloosa, AL 35487 USA; 4 Occupational Health Services, Harry S. Truman Memorial Veterans’ Hospital, Columbia, MO 65211 USA; 5 Center for the Human-Animal Bond, College of Veterinary Medicine, Purdue University, West Lafayette, IN 47907 USA; 6 School of Health Professions, University of Missouri, Columbia, MO 65211 USA; 7 TREE House of Greater St. Louis, Wentzville, MO 63385 USA; 8 School of Nursing and Health Studies, University of Missouri-Kansas City, Kansas City, MO 64108 USA; 9 Cedar Creek Therapeutic Riding Center, Columbia, MO 65201 USA; 10 College of Education, School and Counseling Psychology, University of Missouri, Columbia, MO 65211 USA; 11 Ride-On St. Louis, Kimmswick, MO 63053 USA

**Keywords:** Animal-assisted intervention, Coping self-efficacy, Emotion regulation, Military veterans, Post-traumatic stress disorder, Social engagement, Therapeutic horseback riding, Traumatic brain injury

## Abstract

**Background:**

Large numbers of post-deployment U.S. veterans are diagnosed with post-traumatic stress disorder (PTSD) and/or traumatic brain injury (TBI), leading to an urgent need for effective interventions to reduce symptoms and increase veterans’ coping. PTSD includes anxiety, flashbacks, and emotional numbing. The symptoms increase health care costs for stress-related illnesses and can make veterans’ civilian life difficult.

**Methods:**

We used a randomized wait-list controlled design with repeated measures of U.S. military veterans to address our specific aim to test the efficacy of a 6-week therapeutic horseback riding (THR) program for decreasing PTSD symptoms and increasing coping self-efficacy, emotion regulation, social and emotional loneliness.

Fifty-seven participants were recruited and 29 enrolled in the randomized trial. They were randomly assigned to either the horse riding group (*n* = 15) or a wait-list control group (*n* = 14). The wait-list control group experienced a 6-week waiting period, while the horse riding group began THR. The wait-list control group began riding after 6 weeks of participating in the control group.

Demographic and health history information was obtained from all the participants. PTSD symptoms were measured using the standardized PTSD Checklist-Military Version (PCL-M).

The PCL-M as well as other instruments including, The Coping Self Efficacy Scale (CSES), The Difficulties in Emotion Regulation Scale (DERS) and The Social and Emotional Loneliness Scale for Adults-short version (SELSA) were used to access different aspects of individual well-being and the PTSD symptoms.

**Results:**

Participants had a statistically significant decrease in PTSD scores after 3 weeks of THR (*P* ≤ 0.01) as well as a statistically and clinically significant decrease after 6 weeks of THR (*P* ≤ 0.01). Logistic regression showed that participants had a 66.7% likelihood of having lower PTSD scores at 3 weeks and 87.5% likelihood at 6 weeks. Under the generalized linear model(GLM), our ANOVA findings for the coping self-efficacy, emotion regulation, and social and emotional loneliness did not reach statistical significance. The results for coping self-efficacy and emotion regulation trended in the predicted direction. Results for emotional loneliness were opposite the predicted direction. Logistic regression provided validation that outcome effects were caused by riding longer.

**Conclusion:**

The findings suggest that THR may be a clinically effective intervention for alleviating PTSD symptoms in military veterans.

## Background

Therapeutic horseback riding (THR) is defined as a horseback riding program in which the primary goal is rehabilitation [1, 2]. This study examined the effect of THR on post-traumatic stress disorder (PTSD) symptoms in veterans. Changes in coping self-efficacy, emotion regulation, and social and emotional loneliness were also investigated. Our study advances the empirical exploration into THR as a form of rehabilitation for veterans who have PTSD.

### Social cognitive theory and self-efficacy

Our study was designed using the conceptual framework of social cognitive theory (SCT). SCT explains psychosocial determinants of behavior in terms of triadic reciprocal causation (person, behavior/outcome, environment) [3]. In this transactional view of self and society, personal factors, such as cognitive, affective, and biological events; behavioral patterns; and environmental events, operate as interacting determinants that influence each other.

According to Bandura, in SCT, a major factor of motivation, affect, and behavior is self-efficacy. Persons have a level of confidence, known as perceived self-efficacy, that influences behavior [3]. The actual performance of a behavior (in this case coping) in a specific situation is highly related to the perception that an individual has the ability to perform the behavior. The more strongly self-efficacy is perceived, the more active and persistent are the individual’s efforts toward the behavior.

Bandura [3] noted that depression and social support are two key pathways that impact self-efficacy. Depression negatively influences the individual’s ability to control life stressors. Aspirations are not achieved, and depression is potentiated. Additionally, an inability to develop and maintain social relationships and support contributes to depression and lowers self-efficacy. In our study, perceived coping self-efficacy was veterans’ perceived ability to successfully respond to unforeseen events.

### Post-traumatic stress disorder in veterans

PTSD is an anxiety disorder that occurs after exposure to a life-threatening event or injury [4]. PTSD is marked by four symptom domains: re-experiencing (i.e., flashbacks), avoidance, changes in beliefs and feelings, and hyperarousal. Estimations of the percentage of the more than 23 million veterans who experience clinically significant PTSD symptoms per year vary by service era and are estimated to be in the range of 11%–20% from Operation Enduring Freedom/Operation Iraqi Freedom/Operation New Dawn, 12% from the Gulf War, and 15% from the Vietnam war (30% in their lifetime) [4, 5]. While these numbers are significant, PTSD is likely to be under-reported due to stigma, making these percentages lower than might be accurate among those who have served.

PTSD has been associated with poor quality of life and increased use of health care services, and a variety of co-morbid physical and psychological conditions [6]; most notably depression [7, 8] and substance misuse and addiction to drugs and alcohol [9, 10]. Emotional withdrawal and numbing is common among men, while higher arousal, lack of control, and self-persecution occurs among women [11]. Greater combat exposure has been associated with more PTSD symptoms and poorer readjustment [11].

Research suggests that a common coping response to PTSD among veterans is excessive alcohol use [12–15]. Furthermore, veterans’ attempts to cope with PTSD symptoms through alcohol use may further magnify the challenges of reintegrating into post-deployment life. PTSD, depression, and substance use disorders are associated with a variety of family problems, including marital distress, domestic violence, poor parenting, and a variety of behavioral health problems for children [16]. These consequences place spouses and children at increased risk for their own behavioral health concerns [17, 18].

Additionally, fears of stigma may prevent veterans with PTSD from admitting to symptoms, seeking assistance, or following medical advice. PTSD and chronic pain are commonly comorbid, with each reinforcing and exacerbating the effects of the other [19]. Veterans diagnosed with PTSD were found to have greater psychiatric comorbidity and physical and emotional well-being limitations than those without a PTSD diagnosis [20]. The strong association of PTSD with medical co-morbidities heightens the need to address this disorder as early as possible to lessen demands for VA medical services as veterans age and the disorders they experience become chronic [21].

Cognitive behavioral therapy (CBT) is often a recommended treatment for veterans [22, 23]. Cognitive techniques generally target extinction of conditioned emotional responses by challenging distorted beliefs that result in maladaptive appraisals contributing to the maintenance of PTSD [24]. Behavioral techniques are used to habituate or extinguish stimuli associated with memories of traumatic experiences by presenting a feared stimulus until the fear, anxiety and related problems are reduced [22, 25]. In addition to CBT, positive stress reduction, social support and coping strategies are needed if PTSD symptoms are to be managed effectively. Finally, social support has been found to reduce the negative effects of life events and to positively affect the perception and interpretation of such events [26, 27]. Two meta-analyses showed that strong perceived social support was associated with fewer PTSD symptoms [28, 29].

Despite empirical evidence of the prevalence and potential negative impact of PTSD on veterans, research that examines innovative interventions is scarce. Experts have advocated for research on complementary and alternative therapies (i.e., those used in conjunction with or those which are not considered to be conventional therapies) [30–32]. Animal-assisted interventions are a unique form of complementary and alternative therapy based on human-animal interaction [33]. The International Association for Human-Animal Interaction Organizations has established standards for ethical implementation of animal-assisted interventions [34]. One important avenue to explore for treating PTSD may be human-animal interaction, and specifically, interventions involving horses.

### Therapeutic horseback riding and treatment of PTSD

Clinically, THR is a standardized horse riding program for people with disabilities in which the primary goal is their rehabilitation [1, 2]. THR has been implemented in adults and older adults with a variety of physical impairments [35–37] as well as defined physical and psychological disorders. THR interventions have been designed for individuals with multiple sclerosis [38–40], spinal cord injury [41], spinal stenosis [42], mental retardation [43], and traumatic brain injury (TBI) [44]. Positive psychological, physical and social outcomes have been documented with adults in other THR studies. Psychological improvements due to THR include increased self-efficacy, motivation, and courage [41–44], reduced psychological distress [44], and enhanced psychological well-being [37, 44]. Social benefits include improved social involvement [38]. THR psychological and social benefits may be important factors to facilitate veterans’ coping with PTSD symptoms.

Physical benefits of THR include improved sitting posture [37], motor function [45], postural balance [35, 38], decreased muscle tension [38, 41], improved balance and gait [42], and reduction of pain [38, 46]. Preliminary evidence suggests that PTSD and other anxiety/depression related mental health symptoms may also decrease with physical activity (PA) [47, 48]. PA occurring during THR may be a potential positive coping strategy for veterans with PTSD. In THR, the rider experiences the horse’s stride, using core strength to remain erect, making horseback riding not merely a passive experience, but also a PA. THR in a class setting may foster social support and enhance the veterans’ willingness to do other PA.

There are limited studies involving THR in the treatment of PTSD or TBI. One important case study of a 44-year-old veteran with a spinal cord injury demonstrated both functional improvements, including increased regular PA, along with increases in self-esteem, self-control, and sense of independence [49]. Veterans who participated in an equine assisted learning program (i.e., horses used to promote cognitive reframing and mindfulness) reported that the program had very positive benefits on their PTSD symptoms and coping skills [50]. Veterans who took part in a THR program reported greater communication skills and self-awareness and self-esteem [51]. While these studies provide veteran reported evidence of THR, none employed widely used and standardized instruments to measure outcomes. A single study was identified which showed quantitative decreases in depression and improvements in reported physical health due to a 24-week THR program [52]. However, this exploratory study lacked a control condition, making effectiveness of THR difficult to evaluate.

Horses and THR have been previously used in treating PTSD; however, there are no randomized controlled trials studying the effectiveness of horses in reducing levels of PTSD [53]. THR may be a beneficial activity to reduce PTSD symptoms in veterans and also increase PA, reduce stress, enhance coping self-efficacy, and provide social support. Previously, THR participants have characterized horses as accepting and nonjudgmental [54]. Our specific aim was to test the extent to which participation in a 6-week THR program (riding once per week) was associated with improvements in the primary outcomes of PTSD symptoms and coping self-efficacy as well as in the secondary outcomes of emotion regulation and social and emotional loneliness. Participation was hypothesized to be associated with a decrease in PTSD symptoms, increases in coping self-efficacy, emotional regulation, and a decrease in social and emotional loneliness among veterans. Veterans assigned to the waitlist control group were not expected to have any changes in outcome measures during the 6-week waitlist control period. We selected the waitlist control design to enable all veterans to experience THR, and also to accommodate the capacity of the THR facility. The number of sessions attended in the 6-week THR program was predicted to be associated with improvements in all primary and secondary outcome measures.

## Methods

### Participants

The study had the approval of the VA Research and Development Committee at the Harry S. Truman Memorial Veterans Hospital, the University of Missouri Health Sciences Institutional Review Board and the Animal Care and Use Committee of the University. All participants completed the VA Research Consent Form, which had been approved by the Health Sciences (HS) Institutional Review Board(IRB). Fifty-seven veterans were assessed for eligibility. Nineteen veterans were excluded (13 did not meet inclusion criteria, and 6 for a variety of other reasons). Our primary means of recruitment occurred through letters and postcard invitations. Two invitations were mailed to veterans identified through VA electronic medical records as having met the inclusion criteria of a diagnosis of PTSD, or PTSD and traumatic brain injury (TBI), and who lived within a 50-mile radius of the THR sites. Veterans were also recruited through referrals from VA clinicians and by advertising the study throughout the VA hospital. These veterans contacted the study office to volunteer to participate.

Other inclusion criteria were: age 18 years or older, no longer in active military service (including reserves), weight of 220 pounds or less, able to walk at least 25 ft without the assistance of a person, and willing to interact with and ride a horse. The weight limit of veterans in our inclusion criteria was determined because the horses could not accommodate heavier participants.

All horses working at the riding center were eligible to participate in the study if they were able to accommodate a veteran of up to 220 pounds. The horses that worked in our study were selected by the Professional Association of Therapeutic Horsemanship (PATH)-certified riding instructor for their fitness and experience of being ridden by adults. As part of the ethics approvals, the VA Research and Development Animal Component of Research Protocol (ACORP) involved a visit by a VA-affiliated veterinarian to the riding centers to verify the welfare and husbandry conditions for the horses.

**Fig. 1:**
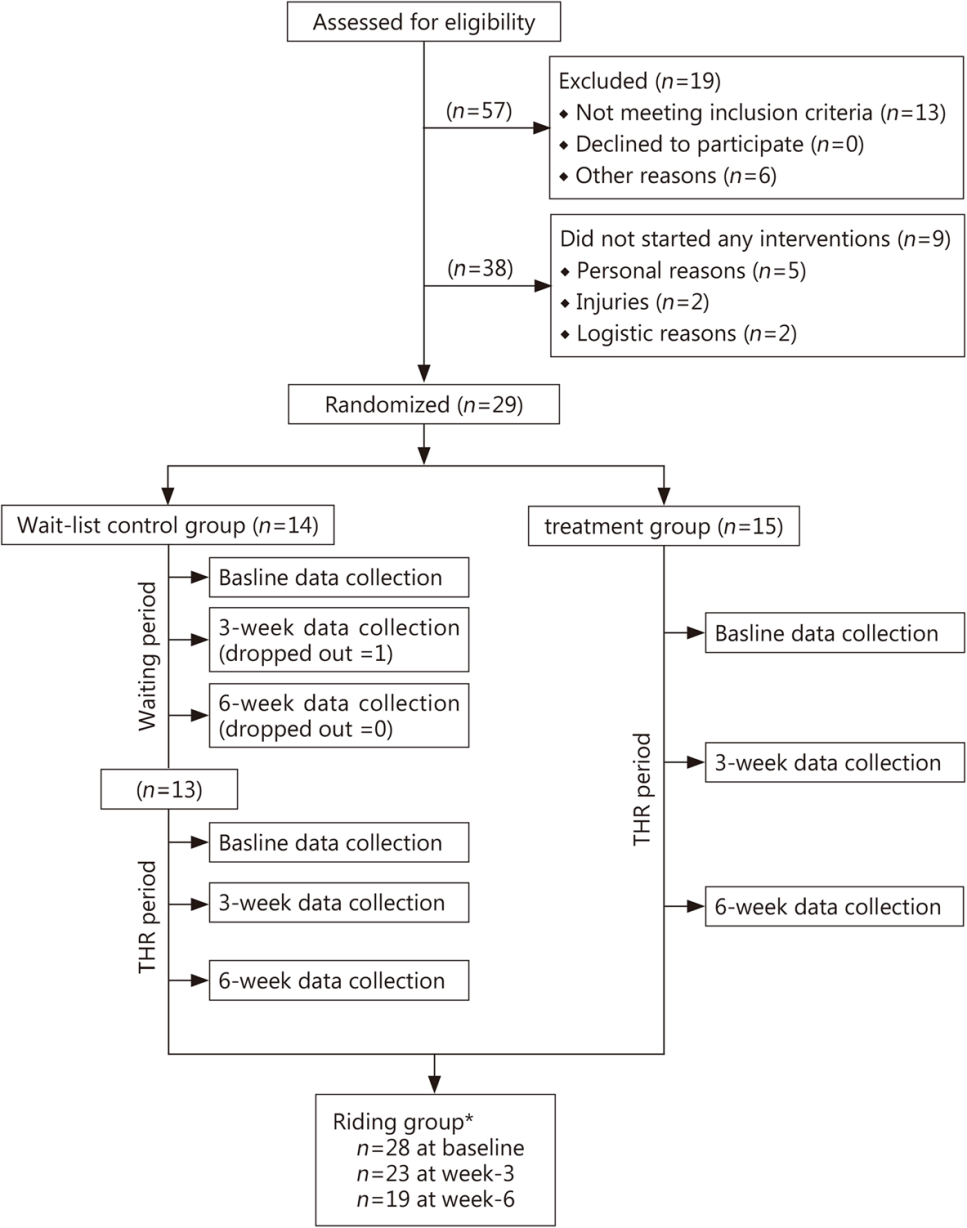
Participant flowchart. *The Riding group data comprise the data of the waitlist control group collected at 0-, 3-, and 6-weeks after THR following the  6-week waiting period and those of the treatment group collected at 0-, 3-, and 6-week after THR

Among 38 participants who were enrolled in the study, 9 participants did not start the THR program due to several reasons (personal matters, *n* = 5; injuries, *n* = 2; logistic issues, *n* = 2). All participants were aware that they would be assigned to one of the two groups prior to providing informed consent. They were randomly assigned to either the riding group (*n* = 15) or a wait-list control group (*n* = 14) based on their identification number (Fig. 1). The wait-list control group experienced a 6-week waiting period, while the riding group began THR. The wait-list control group began riding after 6 weeks of serving in the control group (*n* = 13). When they converted to the treatment group, another set of baseline data was collected from them. This increased the baseline treatment group data to 23 participants. For the 3-week data, there was also data from 23 participants. However, in the 6-week data, due to attrition, there was data from only 19 participants. This waitlist control design has been successfully employed in an equine program research with 131 adolescents in an 11-week equine facilitated learning program [55]. All participants took part in the 6-week THR program. Riding center staff were not aware of who had been assigned to either group.

All participants provided written informed consent, and their primary health care providers gave written assent for their participation in the THR program. To facilitate retention and minimize the attrition of study participants, a strong study identity was created using regular study staff contact with participants and by giving t-shirts to participants depicting study-specific logos. Despite best efforts, attrition occurred; 29 veterans participated in the THR. Attrition occurred for a variety of reasons: 2 participants were injured between enrollment in the study and before the THR classes began; 2 participants no longer replied to telephone calls or emails; 1 did not want to provide a reason for discontinuing participation; 1 cited mistrust of government programs; 1 moved, and 1 said he was not yet ready to be in public. Nine participants who began the THR class did not finish the session, 5 participants discontinued due to family commitments or changes in their schedules (e.g., childcare responsibilities, illness of a family member, or employment), 3 participants no longer responded to phone calls or emails, and 1 participant stopped participating after one riding session because she perceived that the horse she was matched with did not like her. Study staff did not observe any overt negative behavior in the horse, and it was successfully matched with another veteran in the next round of THR sessions. The veteran was offered a different horse but she chose to discontinue the study instead. There were no adverse events (e.g., falls from a horse, biting, kicking, or injuries) during any riding session. The class coordinator had a cellular telephone with a designated toll-free number that participants could call 24-h a day for the duration of the study. This phone number was specifically provided in case participants experienced any injuries or pains due to, or had concerns or questions about the study. No phone calls were received addressing injuries or pains; the phone number was exclusively employed regarding scheduling issues.

### Materials

Demographic and health history information was obtained from participants via investigator-developed questionnaires that were used successfully in previous studies [56]. The demographic questionnaire asked participants’ age, gender, race, marital status, years of education and horseback-riding history. The health history questionnaire included a list of common health problems, asked about drug, alcohol, caffeine and tobacco use, as well as complementary therapies that may be associated with changes in self-efficacy such as yoga [57], massage, meditation, mindfulness [58], biofeedback, acupuncture, and prayer. The health history questionnaire also asked participants to rate their pain during the current week on a 0–10 scale with 0 meaning no pain and 10 meaning the worst pain ever experienced. The demographic and health history questionnaires were administered once at baseline.

PTSD symptoms were measured using the PTSD Checklist-Military Version (PCL-M). The PCL-M is a self-report measure of the 17 DSM-IV symptoms of PTSD asking about problems in response to “stressful military experiences” [59]. Study participants were asked to rate how much they were “bothered by that problem in the past month.” Items are rated on a 5-point Likert scale ranging from 1 (‘not at all’) to 5 (‘extremely’), with higher total scores indicating more PTSD symptoms experienced. A total summed score (possible range of 17 to 85) is obtained, and a score of at least 50 is indicative of a PTSD diagnosis [60]. The PCL-M has been found to have strong internal consistency (0.94–0.97), test-retest reliability (0.97 over 3 days), concurrent validity (0.77–0.93), and diagnostic efficiency with a sensitivity of 0.82 and specificity of 0.84 [60].

The Coping Self Efficacy Scale (CSES) is a 26-item 11-point analogue scale assessing individuals’ perceived ability to cope with life’s challenges or threats by using problem-focused coping, stopping of unpleasant emotions and thoughts, and getting support from family and friends [61]. Higher scores indicate greater perceived coping self-efficacy (possible range of 0 to 260). Participants were asked to rate how well they believed they could perform behaviors important to adaptive coping. The instrument has strong internal consistency (0.80–0.91) and test-retest reliability (0.40–0.80) [62].

The Difficulties in Emotion Regulation Scale (DERS) contains 36 items measuring the “modulation of emotional arousal; awareness, understanding, and acceptance of emotions; and ability to act in desired ways regardless of the emotional state” and is scored on a 5-point Likert-type scale ranging from 1 (‘almost never’) to 5 (‘almost always’) [63]. Higher scores are indicative of greater difficulties with emotion regulation (possible range of 36 to 180). The DERS was found to have a strong internal consistency (0.93) and tested well against the Negative Mood Regulation Scale (−0.69) [63].

The Social and Emotional Loneliness Scale for Adults-short version (SELSA) consists of 15 items assessing emotional, family, and romantic loneliness rated on a 7-point Likert-type scale ranging from 1 (‘strongly disagree’) to 7 (‘strongly agree’) [64]. Higher scores signify greater perceived loneliness (possible range of 15 to 105). The scale was found to have an internal consistency ranging from 0.87–0.90, to be significantly correlated with the long-established UCLA-Loneliness Scale, and has been extensively tested against other established measures [64].

### Procedures

THR classes were held at a Professional Association of Therapeutic Horsemanship (PATH)-Accredited Riding Center study site. An occupational therapist conducted assessments on each participant to ascertain their needs to ensure safety during THR and to identify the appropriate horse for each veteran. The veterans rode the same horse for the entire study period. The facility staff matched each veteran with a horse based on physical criteria and the veteran’s expressed preferences. Baseline data collection, which took approximately 40 min to complete, occurred before any participant began the THR sessions. Subsequent data collection (which required 15–20 min to complete) occurred 3 times for participants randomly assigned to the wait-list control group: at baseline, week 3 of THR, and week 6 of THR. Data collection occurred 5 times for participants assigned to the wait-list control group-control period: at baseline (6 weeks before the start of the THR), control period week 3 (3 weeks before the start of the THR), and control period week 6 (which also served as the THR baseline), as well as THR weeks 3 and 6. Table 1 depicts the instrument administration intervals.

**Table 1:** Data collection schedule

Item	Wait-list control group					Riding group		
	Waiting period			Riding period		Baseline	Week 3	Week 6
	Baseline	Week 3	Week 6	Week 3	Week 6			
Demographic questionnaire	X					X		
Health history	X					X		
Coping Self Efficacy Scale	X	X	X	X	X	X	X	X
PTSD Checklist-Military Version	X	X	X	X	X	X	X	X
Difficulties in Emotion Regulation Scale	X	X	X	X	X	X	X	X
Social and Emotional Loneliness Scale for Adults-short Version	X	X	X	X	X	X	X	X
Riding diary (completed weekly)		X	X	X	X		X	X

X means data collected at that time point

One riding program took place in an indoor arena (4 participants) and the rest took place in a covered, outdoor arena. For the latter, inclement weather was an issue resulting in class cancellation. The riding season began in mid-March and finished at the end of October. During the hottest months (July and August), for the safety of the riders and horses, classes were cancelled twice due to extreme heat and humidity.

Participants attended THR classes once per week for 6 weeks in accordance with the systematized THR curriculum developed by the research team, which included 2 occupational therapists and 2 PATH-certified riding instructors. The THR sessions were conducted by a PATH-certified riding instructor and supervised by an occupational therapist. Horses were led by a riding center volunteer. Side-walkers are used in THR to ensure the safety of participants and facilitate balance if necessary by walking next to the rider at both sides of the horse. Participants were allowed to “fire” their side-walkers beginning week three of our THR curriculum if the riding instructor deemed their progress sufficient.

During the THR sessions, veterans learned basic horsemanship skills and completed tasks on horseback. THR classes consisted of grooming and interacting with the horse before riding, applying the riding tack to the horse, then riding with a horse leader and two side walkers to ensure safety. Each session consisted of the following successive elements: Welcome to the Barn, Grooming and Safety, Mounting, Lesson (warm up exercises, riding exercise, and cool down), and Dismount/Closure. The length of time for the individual elements changed with each lesson as the riders progressed in their horsemanship. The schedule for the first class included: 10 min for Welcome to the Barn, 25 min for Grooming and Safety, 10 min for Mounting, 10 min  for Lesson, and 5 min for Dismount/Closure. The Welcome to the Barn decreased to 5 min and Grooming and Safety to 20 min, while the Lesson increased to 15 min and Dismount/Closure to 10 min (there was no change in time for Mounting). The final class consisted of 5 min of Welcome to the Barn, 10 min of Grooming and Safety, a 30-min Lesson, and 10 min of Dismount/Closure.

### Statistical analysis

Working from a conceptual framework of social cognitive theory, this study sought to determine whether a THR program affects psychosocial determinants of behavior in post-deployment U.S. veterans with a diagnosis of PTSD or PTSD and TBI. Four variables were examined, including PTSD, CSES, DERS, and SELSA, each measured at baseline, 3weeks and 6weeks. The THR program was a randomized wait-list controlled design, in which participants rode a horse or were wait-listed for prescribed times. Demographic data about the participants were also collected. The analysis employed both descriptive and inferential statistics, and was done with SPSS, Version 24 (IBM Corp. Released 2016. *IBM SPSS Statistics for Windows, Version 24.0.* Armonk, NY: IBM Corp.). After screening, there were 29 subjects with complete data. For inferential analyses, relevant statistical assumptions (including normality, linearity, homoscedasticity) were evaluated. Independence of observations was also presumed. Consistent with the study’s design, a repeated measures ANOVA was used on the outcome scores. Also, t-tests on difference scores were calculated: (1) baseline to 3-week, (2) baseline to 6-week, and (3) 3-week to 6-week. For dichotomous outcomes, a logistic regression was employed. Following custom, the *P*-value for determining significance was set at 0.05.

## Results

After customary demographic counts, the primary analysis used was repeated measures ANOVA between factors. Usual assumptions were evaluated, including normality, linearity and homoscedasticity. Each was found to be within acceptable parameters for analysis by variance statistics (i.e., ANOVA). The differences evidencing a significant difference are noted (by convention) with an asterisk and in the table’s footnote.

### Demographics

The sample was comprised of 32 males (84.21%) and 6 females (15.79%). The age was 54.35 ± 12.85(29–73) years. Military service branch consisted of the following (*n* = 38): 17 (44.74%) had served in the Army, 9 (23.68%) in the Marines, 7 (18.42%) in the Navy, 4 (10.53%) in the National Guard, and 1 (2.63%) in the Air Force. One participant declined to divulge this information. The average number of deployments was 1.79, ranging from zero to 10.

### Instrument performance

Internal consistency was assessed for each instrument by calculating Cronbach alpha coefficients. This was important to ascertain the reliability of the instruments with the military veteran population. A value above 0.7 was used as the criterion for acceptable interpretations [65]. The values for each instrument were as follows: PCL-M = 0.737, CSES = 0.868, DERS = 0.831, SELSA = 0.788. These values demonstrate that the study instruments performed well with our participants.

### Primary and secondary outcomes

In order to address the research questions, the analyses included both descriptive and inferential statistics. For descriptive work, frequency counts, frequency descriptions and correlations between relevant variables were calculated. For inferential approaches to data analyses, our primary approach was a repeated measures ANOVA, followed by confirmatory analysis with logistic regression analysis. First, relevant assumptions were evaluated, including normality, linearity and homoscedasticity. Each were within acceptable parameters for analysis by variance statistics (i.e., ANOVA). Table 2 depicts scores for all outcomes measures over the course of the study. Our data show that PCL-M scores decreased considerably at both the 3-week and 6-week data collection intervals during THR. There were no statistically significant changes in the other primary and secondary outcome variables over time (i.e., coping self-efficacy, emotion regulation [mood], and perceived loneliness). However, and importantly, changes in the DERS (which measured emotion regulation) and CSES (which measured coping self-efficacy) scores were in the predicted direction. Changes in the SELSA were opposite the predicted direction (indicating increased loneliness).

**Table 2:** Mean scores for each outcome measure across time

Item	Wait-list control group data			Riding group data		
	Baseline (*n* = 14)	Week 3 (*n* = 13)	Week 6 (*n* = 13)	Baseline (*n* = 28)	Week 3 (*n* = 23)	Week 6 (*n* = 19)
PTSD symptoms	58.36 ± 16.40 (21–75)	57.62 ± 13.15 (31–78)	59.23 ± 14.29 (31–76)	57.72 ± 14.63 (29–79)	53.22 ± 13.8 (28–82)*	47.00 ± 14.67 (26-78)*
Coping self-efficacy	114.43 ± 64.02 5–255)	103.38 ± 61.08 (0–255)	115.00 ± 48.17 (61–236)	115.59 ± 50.55 (20–236)	116.09 ± 50.68 (34–209)	130.21 ± 51.84 (29–234)
Emotion regulation	113.50 ± 27.80 (58–149)	109.46 ± 24.33 (58–147)	110.00 ± 32.07 (43–154)	106.00 ± 29.60 (43–163)	108.65 ± 21.46 (67–147)	99.42 ± 18.31 (70–132)
Social and emotional loneliness	49.35 ± 5.06 (39–57)	52.08 ± 12.47 (27–74)	53.61 ± 8.03 (42–66)	50.38 ± 11.92 (20–72)	53.52 ± 13.70 (28–85)	57.00 ± 10.29 (42–80)

*Change from previous time point statistically significant at *P* < 0.05

The Riding group data comprise the data of waitlist control group collected at 0-, 3-, 6-week after THR following 6-week waiting and those of treatment group collected at 0-, 3-, 6-week after THR

In the riding group data, while riding, veterans had statistically significant decreases in their PTSD symptoms over the 6-week THR program. Symptoms significantly decreased between baseline and week 3, *F*(1,17) = 10.678, *P* = 0.005, and also between week 3 and week 6 of riding, *F*(1,17) = 8.750, *P* = 0.009. Eighteen of the 23 veterans (78%) (one veteran missed the 3-week data collection) who completed data collection at baseline and after 3 weeks of THR showed a decrease in PTSD symptoms, while 18 out of 19 (94.74%) showed a decrease between baseline and week 6.

**Fig. 2:**
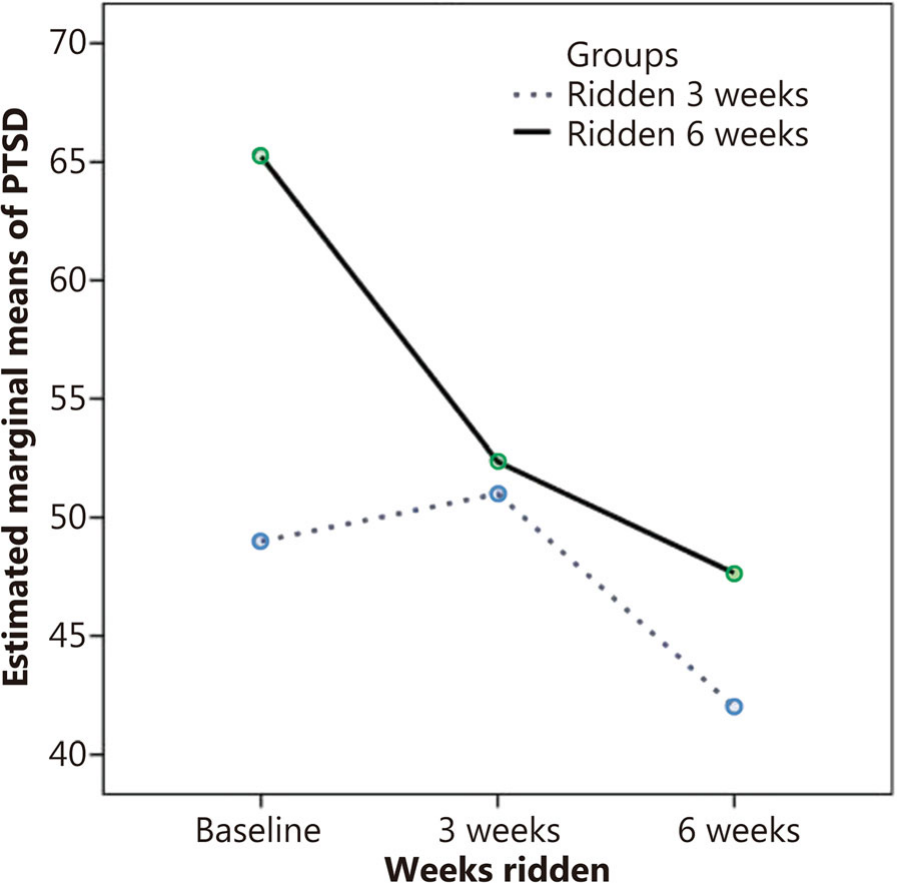
PTSD means of Riding group over time by total number of weeks ridden. The purpose of these analyses was to ascertain whether the significant decrease in PTSD found at 3 weeks of riding was sustained at 6weeks. Each participant (*n* = 19) was tested whether or not they rode for all the 6 weeks

The research design allowed for several meaningful comparisons and contrasts in our data. We were able to construct a variable for the number of weeks that participants rode. We used this variable to compare outcome measures across the 3 data collection points. By this comparison, there were dramatic changes at all 3 data collection points. Globally, the highest level of PTSD symptoms was recorded at baseline, then there was a drop at 3 weeks of riding and a still further drop at 6 weeks of riding. However, the decline in PTSD symptoms was not uniform for all participants. This is shown by the contrast between: 1–3 week riders (dotted line in Fig. 2) and 4–6 week riders (continuous line). Those who rode for more sessions (4–6 weeks) evidenced much larger declines in PTSD scores than those who rode for fewer sessions, and the decline continued with each measurement. The THR intervention’s practical significance is buoyed by the fact that the reduction was evidenced consistently for virtually all participants. Figure 2 displays these findings.

Overall, participants had an 81.8% likelihood of improvement in PTSD levels. Further detailed examination showed that participants had a 66.7% likelihood of having lower PTSD scores at 3 weeks, and an 87.5% likelihood at 6 weeks.

For self-efficacy (CSES scores), the anticipated direction of change is upward, indicating that the individual’s adaptive coping was increasing. As seen in Table 2, the wait-list group had a decline in their success with coping, but both riding groups evidenced increased coping: the shorter riding group (1–3 weeks) did show a decline between the first and second measurement, but then showed a marked increase at the next measurement, while the longer riding group (4–6 weeks) demonstrated consistent CSES increases across all measurements.

By logistic regression, there was 100% correct classification of increased coping, regardless of whether the group was 1–3 weeks or 4–6 weeks. The more the participants rode, the higher their CSES scores were. For the variable DERS, the direction of anticipated change with THR is a decline. The data (Table 2) indicate there was no change with shorter term riding (1–3 weeks), but that the decline as a result of the longer term riding (4–6 weeks) was substantive. As confirmatory information, the logistic regression allowed 100% prediction in correct classification.

For the final outcome variable, the SELSA, the results were less clear than for the other study variables. For both the wait-list and the riding groups, the SELSA scores indicated that social and emotional loneliness increased; however, for the longer riding group (4–6 weeks), the increase was temporary only until the second measurement, whereupon the SELSA scores declined precipitously.

## Discussion

### Primary and secondary outcomes

Our findings need to be interpreted within the clinical context for treatments administered to veterans with PTSD. A 5-point decrease is the minimum threshold used to determine that an individual has responded to a treatment modality [66]. A 10-point improvement is the minimum threshold for determining clinically meaningful improvement [66]. Our findings show that our participants’ mean improvements in PTSD symptoms were 6 points at the 3-week data point and 13 points at the 6-week data point while riding. As such, participants’ PTSD symptoms had beneficially responded to THR after only 3 weeks, and by the end of the THR program, they had definitively achieved a clinically meaningful improvement in PTSD symptoms. Kazdin [67] advocated that the clinical significance of an intervention impacts a person’s functional ability. We conclude that THR shows promise as a beneficial intervention for veterans with PTSD, but did not measure functional ability. Our findings suggest that riding is a constructive activity for reducing PTSD symptoms in our participants and that riding for longer periods of time has a stronger influence than riding for shorter periods of time. Studies examining resultant functional ability would be useful. Kazdin [68] also indicated that the magnitude of change in a dependent variable determines to what extent we may accept causal influence. Others found that PTSD symptoms responded to complementary therapies such as guided imagery [30] or yoga [56]. The fact that 13 of our participants had served in the Vietnam war era, and thus had perhaps been living with PTSD for decades, yet derived a clinical meaningful improvement in their PTSD symptoms from a brief 6-week THR intervention is promising. Our findings provide empirical evidence that THR is effective at improving coping skills and in lessening one’s difficulty with emotional regulation, especially with longer riding interventions.

We recognize the many potentially extraneous variables that may have influenced our findings. The question arises, “What components contributed to the change?” First, we recognize that there is an inherent selection bias in THR research because only participants who were willing to ride a horse sought to enroll. However, we argue that no intervention toward which a person has a negative predisposition will be of benefit to that person; it is not possible to force people to participate in psychosocial interventions that they are unwilling to experience. We do not purport that THR is the intervention of choice for all veterans with PTSD, but only for those willing to ride horses.

Methodologically, a long list of extraneous variables must be taken into account as we interpret our findings. So, the question may be asked, “what components of the THR and the conditions surrounding THR may have contributed to our beneficial findings in PTSD?” For example, driving to and from the stable, indoor versus outdoor riding arenas, and the weather while riding may have influenced the outcomes. It is not possible to isolate these factors. Additionally, the fact that THR consists of many steps including grooming the horse and interacting with it, applying the riding tack to the horse, learning basic horsemanship skills, as well as interacting with the horse leader and side-walkers may have enhanced the participants’ PTSD outcomes. These are all components of THR and cannot be isolated in our study to ascertain their individual effects on our outcomes. It is not realistic that each of these components could be studied separately in a randomized controlled trial; their individual relevance is perhaps less important than the complete THR experience because these components would not naturally occur in isolation. A common criticism of human-animal interaction research is that we cannot be sure that change is caused by only the animal because an animal handler is nearly always involved in such interactions. That is the nature of such interventions, and it would not be possible to study them without handlers. What was done, however, was to objectively study the biophysiologic parameters at each phase of the THR process (e.g., driving to the stable) to better isolate the precise contribution of the actual time on the horse to the changes in the PTSD levels.

Our findings for coping self-efficacy, emotion regulation, and social and emotional loneliness did not reach statistical or clinical significance. The fact that coping self-efficacy and emotion regulation findings moved in the predicted direction was encouraging. We suspect that the diminishing sample size may have limited our power to detect statistically significant changes in these two variables. Alternatively, perhaps a longer THR program would have had a greater impact on these two variables. However, the loneliness findings moved in the opposite direction from our predictions. Although again not statistically significant, this trend warrants further discussion. One potential limitation of the SELSA with combat veterans is that loneliness, which may be associated with guilt and/or shame, is called spiritual or existential loneliness [56]. Another limitation is that it does not capture experiential isolation, which has been defined as failed inter-subjectivity [69]. Our findings may suggest that more work is needed in the conceptualization and operationalization of loneliness in veterans.

The “firing” of side-walkers was performed once participants felt comfortable and confident in their riding abilities and the THR staff was sure they were able to ride on their own. While this marked increased ability, it also lessened the interpersonal interaction with staff. The veterans in this study expressed that the interpersonal interactions with study staff was one of the benefits of participation. Decreasing the time spent with staff during the class session may have influenced our findings.

On the most fundamental physiologic level, research has provided evidence that basic human emotions rooted in the limbic system generally do not occur in opposition to each other (e.g., fear and affiliation, panic and play) [70]. In our study, veterans interacted with their horses (by grooming, tacking, rewarding-some brought carrots for their horse) and spent quiet time talking with their horses. Other investigators have reported that human touch is an essential factor in the creation of human-horse affiliations [71]. This affiliation may increase the likelihood of veterans experiencing a reduction in anxiety, which is a central component of PTSD.

The fact that we found significant improvements in PTSD but not in self-efficacy raises the issue of the difference between these constructs. PTSD is an anxiety disorder, but self-efficacy reflects a person’s belief or confidence in their ability to act on their environment [3]. Our THR intervention may have enhanced the context for the veterans to gain a skillset that would enhance their self-efficacy (e.g., with horsemanship), and while the scores on the CSES were in the predicted direction (improvement), they did not reach statistical significance. The fact that there was no change in these scores during the control period, but there was a positive change during the riding period would support this notion. Moreover, qualitative findings suggested that the veterans felt that they gained confidence. However, our THR curriculum may not have been sufficiently structured to bring to the veterans’ awareness that they were learning a series of horsemanship skills. Furthermore, self-efficacy has been reported to be context or domain specific [72]. While our participants may have increased their self-efficacy for horsemanship, we did not measure this specifically. This is an important consideration for future THR programs aimed at improving self-efficacy.

Our previous research demonstrated that emotionally stressed adult cancer patients who interacted quietly with a companion animal reported the visiting dog provided them comfort and was a confidant who made them happy and gave them energy [73]. Other research demonstrated that quietly petting a dog was associated with a relaxation effect (lower blood pressure and cortisol levels) [74]. Horses differ significantly from dogs; however, the quiet interaction between people and horses working in THR may produce similar experiences of affirmation and relaxation [75]. As prey animals, horses may be less likely than dogs to compete with humans for leadership.

Veterans with PTSD may experience stigma associated with others’ negative perceptions of them, leading to isolation. Moreover, the anxiety associated with PTSD is known to induce veterans to avoid social contexts, leading to further isolation [51]. Afflictive behavior between person and horse has been identified as a vital component of best practices in veterinary medicine when veterinarians work with horses [71]. It can be argued that THR will be more successful when military veterans also interact quietly and kindly with the horses they are riding. In our study, veterans engaged in this behavior at every session prior to and after riding, which may have positively potentiated the effects of our intervention and is a strength of our design.

Our findings regarding social and emotional loneliness were not statistically significant, and the trends were not in the predicted decreasing direction. The participants expressed sadness that the THR program was ending. For example, one veteran said, “I enjoyed the closeness with the horse—met some very nice people.” Another said, “it was really amazing, I really want to continue. I really will miss Rock [horse’s name]. I am always happy around him and I think he responds well to me also.” It may be that loneliness scores at six weeks were related to the veterans’ anticipation of the completion of the program.

Interactions between the veterans and the riding center and study staff were positive. Other investigators have advocated for an individualized approach to selecting “talkative volunteers” or less talkative volunteers as a match with each veteran’s needs, which may enhance veterans’ THR experience [51]. We attempted to control for possible confounding effects of interpersonal interactions with riding center volunteers and study staff by instructing these individuals to minimize conversational initiation with the veterans. In this way, the veterans chose their level of interaction.

### Veterans’ perceptions of THR

At the beginning of our study, some veterans expressed reluctance to participate on receiving our first invitation letter. They were more receptive to the subsequent postcard that we sent with the study logo on it. One gentleman who was a Vietnam war veteran said that he did not want to participate, but his wife encouraged him to come. However, after his first session (which occurred the week before the University went on spring break and the THR was also on recess), he thought that it was too bad to have to wait 2 weeks to do this again. This veteran not only completed the study, he expressed interest in continuing to volunteer at the riding center after completion of the study.

Veterans expressed interest in participating in the study to try something new or rekindle a childhood experience. Many had ridden horses as children and recalled this fondly. Fortunately, veterans who wanted to continue THR were able to do so after the study was completed. This occurred via prescriptions for THR facilitated by VA recreation therapists or when the veterans volunteered at the therapeutic riding centers. Volunteering has been found to be a meaningful activity for veterans, particularly those who served in combat [76].

Accessibility to riding centers may be an issue for veterans who wish to participate in THR. One veteran said “I had to drive an hour to and from the horse center”, and we know this was one of the challenges of the program. THR may be more accessible to rural veterans who may have to travel long distances to reach VA treatment programs. THR is clearly not a replacement for conventional therapies used to treat PTSD, but as a complementary therapy, riding centers may be a readily accessible resource to veterans in rural areas.

### Limitations

Our sample size was small, which limited our power to detect changes in the dependent variables. Nevertheless, we did find statistical and clinical significance in lowering PTSD symptom levels. The sample size was limited by the local VA requirement that we could only recruit participants being treated at the VA through which the study was approved. Originally, we partnered with two THR centers in the St. Louis area. Only 4 of the 38 participants who were treated at the Columbia, Missouri VA lived close enough to these two riding centers to be able to travel there.

The study was logistically complex to implement because of the need to balance VA requirements and approvals, the riding center schedules, university calendars, and the busy lives of the participants. One previously mentioned veteran drove 1 h, while others drove 40 min to attend the classes, which occurred during normal business hours to accommodate the riding center. Three volunteers were needed per veteran (2 side walkers and a leader). The volunteers are essential to the daily operation of the riding center, increasing the complexity of scheduling. We required an Occupational Therapist and a PATH-certified riding instructor to administer the detailed riding curriculum. We also had study staff present to monitor fidelity to the riding curriculum, oversee data collection, and monitor participant safety. The staff also phoned the veterans each week to confirm their attendance at each session or apprise them of changes in class schedule due to inclement weather.

We did not use a longitudinal follow-up. It would have been helpful to identify to what extent the PTSD symptoms remained lower after time passed since the riding program ended. Furthermore, the length of our THR program may have been too short. Lanning and Krenek [51] used a 24-week THR program in their study, which demonstrated improvements in veterans’ physical health and depression levels. However, for PTSD symptoms, our findings demonstrated that 3 weeks was an effective intervention length and that even more improvement was noted at 6 weeks.

### Implications

Those planning THR programs would do well to address some of the lessons we learned from our study. While our findings showed beneficial outcomes in PTSD at the 3-week measurement, longer participation (six weeks) was beneficial for self-efficacy and emotion regulation. We identified steps that could be taken to minimize veterans’ attrition. For example, assisting with transportation to the riding center and providing child care during the classes may be beneficial. Climate controlled riding centers would maximize comfort for participants, volunteers, staff and horses, while minimizing the likelihood of having to cancel a class session. Horses able to carry riders weighing more than 220 pounds would increase the numbers of veterans who can participate. In addition, provision of THR class scheduling options may decrease the number of classes missed by veterans due to scheduling conflicts.

## Conclusions

Our findings have honed the existing knowledge base on THR, a beneficial intervention for veterans with PTSD, by identifying a clinically meaningful dosage of THR. A 3-week THR program was effective, and a 6-week program produced clinically significant outcomes in PTSD levels. Older veterans, such as the majority in our sample, may have been diagnosed with PTSD decades ago; THR may be particularly promising for them. It may be important for health systems to recognize such promise by supporting THR as a reimbursable complementary therapy.

## Abbreviations

ACORP: Animal component of research protocol
CBT: Cognitive behavioral therapy
CSES: Coping self efficacy scale
DERS: Difficulties in emotion regulation scale
PA: Physical activity
PATH: Professional association of therapeutic horsemanship
PCL-M: PTSD checklist-military
PTSD: Post-traumatic stress disorder
SCT: Social cognitive theory
SELSA: Social and emotional loneliness scale for adults-short
TBI: Traumatic brain injury
THR: Therapeutic horseback riding
